# Acoltremon and the TRPM8 frontier: a new chapter in dry eye disease therapy

**DOI:** 10.1097/MS9.0000000000003763

**Published:** 2025-08-28

**Authors:** Fakiha Sultana Malik, Fatima Naveed, Aleeza Khan, Mahnoor Fatima

**Affiliations:** a Nishtar Medical College, Nishtar Medical University, Multan, Pakistan; b Shaheed Ziaur Rahman Medical College, Rajshahi Medical University, Bogura, Bangladesh

Dry eye disease (DED) is a complex and evolving condition that affects the ocular surface and surrounding structures, often leading to symptoms like dryness, irritation, and fluctuating vision. It stems from tear film instability and is frequently accompanied by inflammation, damage to the ocular surface, and in many cases, neurosensory disturbances[1].The mechanisms are complex, and the burden is widespread. Current studies estimate that approximately 16.4 million Americans or 6.8% of the adult population have been formally diagnosed with DED. Alarmingly, an additional 6 million adults are believed to experience symptoms consistent with DED without receiving a diagnosis, placing the total estimated burden at nearly 9.3% of the adult U.S. population[2]. These figures highlight the urgent need for both better awareness and more effective therapeutic strategies. This article was written in accordance with the TITAN guideline checklist 2025[3].

DED is now one of the most common yet overlooked health issues, driven less by contagion than by modern lifestyle factors. Affecting anywhere from 11% to 30% of people worldwide, it is especially common among older adults, postmenopausal women, contact lens wearers, and individuals who spend long hours in front of screens[4]. With the rise of digital life, blinking rates have dropped and ocular surface integrity is compromised. Once linked to aging, DED now increasing affects younger population due to *Computer-Vision Syndrome*[5]. At the same time, as the global population ages, tear production naturally declines, and eye inflammation becomes more common which further adds to the already alarming situation. Additionally, our exposure to pollution, dry indoor air, and long hours in artificially lit environments worsens dry eye burden worldwide. Despite its high prevalence, DED is frequently underdiagnosed or misattributed to nonspecific ocular discomfort[6]. But this mix of modern habits and environmental stressors has made DED a condition with significant public health implications.

A new era in dry eye disease treatment begins with the FDA approval of Acoltremon, sold under the name TRYPTYR (0.003% ophthalmic solution). It is the first treatment of its kind that targets TRPM8 (Transient Receptor Potential Melastatin 8) receptors, providing quick relief, sometimes as early as day 1. TRYPTYR is available in convenient, single-use vials, with a recommended dosage of one drop in each eye, twice daily. Alcon plans to launch TRYPTYR in the U.S. during the third quarter of 2025 and aims to expand to other markets in the future[7]. Its FDA approval introduces a promising new option that could revolutionize dry eye treatment.

The TRPM8 receptor, located in the sensory nerves of the cornea and eyelid, specifically in the ophthalmic branch of the Trigeminal nerve, is a type of polymodal, calcium-permeable, nonselective cation channel which functions as a cold and menthol sensor in the body. When stimulated by tear film evaporation (which produces a cooling effect), these receptors open, causing an influx of calcium and sodium ions in the sensory neurons, leading to their depolarization and the production of an action potential that is interpreted by the brain as a “cooling” or “dryness” effect. This causes reflex production of a tear film, regulating moisture on the ocular surface.^[8-10]^

Acoltremon, a TRPM8 receptor agonist, works by targeting these receptors as a cold-sensing modulator, helping boost natural tear flow while also giving a soothing, cooling sensation that relieves dry eye symptoms^[8,9]^. Additionally, Acoltremon’s cooling effect may offer relief to patients dealing with neuropathic eye pain, a condition with few effective treatment options^[10,11]^. Hence, Acoltremon may prove beneficial for increasing tear production as well as reducing the ocular discomfort in the treatment of DED[12].

The FDA approved Acoltremon on the basis of two successful Phase 3 prospective, randomized, double-masked, vehicle-controlled, parallel-group, multicenter studies. In the COMET-2 and COMET-3 trials, patients who had signs and symptoms of dry eye disease at both the screening and baseline visits, along with a corrected visual acuity of +0.70 LogMAR or better in both eyes as part of the inclusion criteria, received either 0.003% AR-15512 ophthalmic solution or its vehicle control, administered as one drop in each eye twice daily for 90 days. Administration of Acoltremon demonstrated a statistically significant increase in the percentage of subjects who achieved a ≥10 mm improvement in their 5-minute unanesthetized Schirmer Tear Test scores by Day 14, compared to baseline^[13,14]^. Among the 930 total dry eye patients, 51% reported instillation site burn and injury, and 98% reported it as mild in severity and less than 1% of patients discontinued therapy due to burning or stinging[7].

In the preceding phase 2b study, 0.003% Acoltremon showed sustained improvements in unanesthetized Schirmer score (Days 1 and 14, p < 0.0001), ocular surface staining (Days 14 and 84, p ≤ 0.0365), and hyperemia (Day 84, p < 0.0215). Significant improvements were also noted on SANDE (Days 14, 28, and 84, p ≤ 0.0254), ODS-VAS (Day 84, p = 0.0281), Eye Dryness-VAS (Day 84, p = 0.0302), and multiple QoL measures (Days 14, 28, and 84, p < 0.05). The group receiving AR-15512 did not significantly differ from the placebo group in the co-primary end points. 0.003% Acoltremon was well tolerated and only mild instillation site burning or stinging in ≥2% of subjects[12].

These results from the COMET studies suggest that the 0.003% Acoltremon ophthalmic solution addresses the limitations of current dry eye prescription options. In clinical trials, improvement in unanesthetized Schirmer scores was observed as early as Day 1 post-drop, accompanied by significant reductions in discomfort scores and a favorable safety profile. All these outcomes for the first-in-class TRPM8 receptor agonist open the door to new perspectives in the treatment of the rapidly increasing number of patients with dry eye disease[15].

Regarding the safety of the drug, no serious side effects have been reported. However, in about 50% of the participants, the most commonly reported side effect during clinical trials with Acoltremon was instillation site pain[15].

Despite encouraging results from COMET-2, COMET-3, and earlier phase trials, several important questions remain. Most notably, the long-term safety and efficacy of Acoltremon beyond the 90-day trial window remain untested, particularly for patients with chronic DED who require extended therapy. While the trials showed statistically significant improvements in Schirmer scores and symptom scales, real-world outcomes such as visual performance and daily functioning were not primary endpoints and need further investigation. Another key limitation is the heterogeneity of DED itself; it remains unclear which subtypes (e.g., evaporative vs aqueous-deficient, or neuropathic vs inflammatory) may derive the greatest benefit from TRPM8-targeted therapy. Additionally, although tolerability was generally good, the high rate (51%) of instillation site discomfort even if mostly mild raises questions about adherence in broader, more diverse populations. Future studies should include head-to-head comparisons with existing treatments to better define Acoltremon’s place in current clinical practice.

Existing pharmacologic treatments for DED such as cyclosporine (Restasis), lifitegrast (Xiidra), and short-term corticosteroids primarily target inflammation and often require prolonged use before clinical benefit is achieved. These agents may not adequately address neurosensory dysfunction, a core component of many DED cases[16]. Moreover, adherence remains a significant challenge due to the delayed onset of action, ocular irritation, and high cost. In contrast, Acoltremon’s unique mechanism of stimulating TRPM8 receptors allows for faster tear production and symptomatic relief, with a safety profile that may support better patient adherence.

Looking ahead, Acoltremon opens promising avenues not only for symptom control but also for advancing our understanding of sensory regulation on the ocular surface. Its potential role in managing neuropathic ocular pain, a domain where current therapies fall short represents an important research frontier. Future studies may explore its use in combination regimens or as a first-line agent in specific DED phenotypes. Over time, its integration into personalized treatment strategies based on disease subtype, severity, and patient lifestyle could significantly reshape how clinicians approach dry eye management.

Although cost data are pending, Acoltremon’s twice-daily dosing, early symptomatic improvement, and dual impact suggest it may be cost-effective by reducing treatment complexity and improving quality of life. Ongoing real-world studies and post-marketing data will be crucial in determining its broader value within healthcare systems.

The approval of Acoltremon is more than a therapeutic milestone it reflects a shift toward more mechanism-specific, patient-centered approaches in ophthalmology. As digital lifestyles and aging demographics continue to drive the global burden of dry eye disease, such innovations represent a timely and essential advancement in addressing the increasing burden of DED.

## Data Availability

Data sharing is not applicable to this article.

## Peer and provenance statement

Not commissioned; peer review pending.
